# Attitudes Toward Digital Planetary Health Before and After a Lecture Series on Environmental Awareness and Future Visions: Repeated Cross-Sectional Survey Study Regarding Twin Transformation

**DOI:** 10.2196/89259

**Published:** 2026-08-04

**Authors:** Julia Nitsche, Theresa Sophie Busse, Sonja Schmalen, Ina Otte, Jan Peter Ehlers

**Affiliations:** 1 Department of Didactics and Educational Research in Health Science, Faculty of Health, Witten/Herdecke University, Alfred-Herrhausen-Straße 50, Witten, North Rhine-Westphalia, 58455, Germany, 49 02302 926 ext 78625; 2 Junior Professorship for Digital Health, Faculty of Health, Witten/Herdecke University, Witten, North Rhine-Westphalia, Germany; 3 Health for Future, Deutsche Allianz für Klimawandel und Gesundheit, Berlin, Germany; 4 Department for Health Care Research, Institute for Diversity Medicine, Ruhr University Bochum, Bochum, North Rhine-Westphalia, Germany

**Keywords:** education, digital medicine, technology-enhanced learning, digital competencies, technology, planetary health

## Abstract

**Background:**

The focus on sustainability in university teaching and education about the climate catastrophe is constantly increasing and is essential for creating change. The chances offered by digital technological innovations are decisive in spotlighting planetary health education. Alongside welfare economies and social movements, they are among the areas with great potential to drive sustainable development.

**Objective:**

This study aimed to evaluate attitudes toward digital planetary health before and after a fully digital planetary health lecture series. This included assessing environmental health awareness, climate-related health risks, and perceptions of the need to integrate digital and planetary health education into university curricula.

**Methods:**

A repeated, pseudonymous, nonexperimental, cross-sectional online survey was conducted before (T1) and after (T2) a fully digital lecture series on digital planetary health during the 2022-2023 winter term. Because participant matching across time points was unsuccessful, descriptive analyses were conducted at the group level.

**Results:**

A total of 180 participants completed the T1 survey, and 79 participants completed the T2 survey. The mean age in the T1 survey was 27.92 (SD 10.95) years, with a minimum age of 18 years and a maximum age of 77 years. The mean age in T2 was 29.22 (SD 12.78) years, with a minimum age of 18 years and a maximum age of 78 years. In the T2 survey, 70.9% (56/79) of the participants rated it as “(very) important” (mean 5.38, SD 1.15) to incorporate climate-related health impacts and digital health issues into their studies. In the T2 survey, 51.9% (41/79) of the participants stated that the range of educational events on digital health at universities was inadequate. Qualitative responses highlighted perceived benefits of digitalization for collaboration, knowledge dissemination, and health care access, while concerns regarding resource consumption and ethical challenges were also described.

**Conclusions:**

Participants who attended the lecture series perceived digital planetary health as highly relevant to university education. The findings provide exploratory insights into attitudes toward the integration of planetary health and digital health topics into higher education curricula.

## Introduction

The focus on sustainability in university teaching as well as education about the climate catastrophe is steadily increasing [1]. In this context, Rossa-Roccor et al [2] further see academics as having an obligation to take an active role in the sphere of knowledge and action. They call for a commitment to policy change through an increased focus on planetary health; in short, to highlight climate change as a human health problem [2].

A meta-analysis of systematic reviews by Rocque et al [3] on the health impacts of climate catastrophe included 94 articles. They found meteorological and extreme weather events as the most common climate impacts. The most common health impacts were (1) infectious disease; (2) mortality; and (3) respiratory, cardiovascular, or neurological diseases [3].

However, this knowledge is not strongly disseminated among health care professionals (HCPs). Hathaway and Maibach [4] found that while there is a perception among HCPs toward the potential harm of the climate crisis on health, the self-assessed knowledge of HCPs is low. Societal perceptions of the hazards of the climate crisis are also high [4]. This supports the need for education in society at large, and especially for students (in the health professions), who can shape tomorrow’s society and drive individual and political change.

Decisive for the particular focus on planetary health education are the opportunities presented by digital technological innovations. Along with economic prosperity and social movements, these innovations are one of the areas with great potential to drive sustainable development [5]. The joint realization of a transformation toward a society that is both sustainable and digital is often referred to as a twin transformation. It is important that these processes influence each other positively (an integrated path) and do not compete with one another [6].

Against this background, the lecture series *Digital Medicine Goes Planetary Health* (DMgPH) was developed in the 2022-2023 winter semester, focusing on the connections between digital health care and planetary health. Lecturers from Witten/Herdecke University (UW/H; JN and JPE), German Climate Change and Health Alliance (SSch), and Ruhr University Bochum (RUB; TSB) organized and moderated weekly 90-minute sessions in which they themselves, as well as guest lecturers from various areas of digital planetary health, spoke.

Although there has been a clear trend toward activating, participatory, and competence-oriented teaching formats in the discussion on university teaching in recent years, traditional formats such as lectures retain their didactic relevance under certain conditions [7]. The much-discussed change in basic assumptions from teacher-centered to learner-centered formats [8,9] has led to a meaningful shift in focus. Nevertheless, studies show that lectures—especially in digital and interactive formats—can make an independent contribution to higher education [6,10]. The online lecture series used in this project offers a concise example of this: digital access has made it possible to reach a broad, transdisciplinary audience and involve many external experts who would not have been available for small face-to-face seminars [11]. Lecture series not only promote the dissemination of current scientific findings but also enable a multiperspective examination of the complex challenges in the context of digital planetary health [2,5]. The focus here is on encouraging further engagement with the topic and highlighting its relevance [12]. Lectures are the most effective way of achieving this with a large audience, as they help to develop a mutual understanding [13]. A more in-depth examination of the topic should then take place in more learner-centered formats [14].

The aim of this study was to survey the attitudes of participants before and after participation in DMgPH regarding environmental awareness and visions of the future. The following research questions were considered:

- What attitudes and self-reported perceptions regarding climate-related health impacts and digital planetary health were reported by participants before the lecture series?
- How do group-level responses before and after the lecture series differ regarding environmental awareness and perceptions of digital planetary health?

## Methods

### Ethical Considerations

Ethical approval was obtained from the ethics committee of UW/H, Germany (approval code: S-198/2022). Participants did not receive compensation for their participation.

All participants provided informed consent prior to participation. For the online survey conducted via LimeSurvey, participants were presented with detailed study information and were required to confirm that they had read and understood the information and agreed to participate before accessing the questionnaire. Participation was voluntary, and respondents could discontinue the survey at any time without disadvantage.

For the qualitative data collection, participants were likewise informed that their responses would be anonymized and used exclusively for research purposes. They were instructed to provide qualitative responses only if they agreed to the anonymized use of their data for scientific analysis.

### Setting and Lecture Series

The lecture series was also held in conjunction with a lecture series on transformative education in Germany, organized by the Planetary Health Academy, which, however, did not focus on digitalization [14]. DMgPH wanted to include the themes in the curriculum and move away from focusing on the personal footprint in relation to the climate crisis and toward increasing the handprint [15]. The handprint is a concept that focuses on actions and the possibility of changing things as an individual [16].

DMgPH was delivered digitally using Zoom meeting software (Zoom Communications, Inc) during the winter term of 2022-2023. Students at UW/H and medical students at RUB were eligible to receive 2 European Credit Transfer and Accumulation System credits in the DMgPH. This was tracked via logins using personalized links; therefore, there was no need to register in advance with real names on Zoom. Students from other universities were given the opportunity to have their participation in the lecture series confirmed and to submit this to their respective examination offices for credit.

Sessions 1 to 11 were characterized by 1 or 2 keynotes by experts from various fields (eg, hospitals and nongovernmental organizations). Each session was followed by a subsequent discussion (chat or video). Work in breakout sessions, surveys, and encouragement to join discussions were used to improve attendee participation. The lecturers moderated the discussions in turn.

At the 12th session, a bar camp was held. Bar camps are characterized by questions from the participants or concrete input [17] and designed to create learning spaces together with the participants [18] where they meet as equals [19]. In DMgPH, the participants were asked in advance to indicate possible areas of interest on Moodle (Moodle Pty Ltd) or to offer their own sessions in the lecture series. Six sessions were offered in which both the lecturers and other experts presented certain topics with a short keynote speech, and discussion arose through joint work assignments (Table 1).

The final session was designed as a quiz in which questions were asked about all previous dates in the lecture series. The 207 participants had 30 seconds to answer each question. Three winners (for the quickest right answers) were awarded prizes as a pleasant incentive (eg, stickers, socks, and a hip bag).

**Table 1.** An overview of the *Digital Medicine Goes Planetary Health *program in WT22/23^a^.

Session	Topic	Lecturers
1	Introduction to planetary health and digital medicine	JN, TSB, SSch, JPE, and Head of German Climate Change and Health Alliance (KLUG e.V.)
2	Digitization, sustainability and global health	Professor of Witten/Herdecke University and a collaborator of the CO:DINA project [20]
3	The German Sustainability Code and its implementation at the University Medical Centre Hamburg-Eppendorf	Employee of the German Sustainability Code and an employee of the Sustainability Department of the University Medical Center Hamburg-Eppendorf
4	Sustainability and telemedicine Green Health – what saves the world?	Director of the Department for Trauma Surgery, Orthopedics and Sports Medicine at Helios University Hospital Wuppertal Employee from eternalHealth [21]
5	Gamification for planetary health	Staff of the German Foreign Office and Leuphana University
6	Democratization of health and value-based healthcare	Oxford University collaborator and Patients4Digital contributor
7	Legal perspective on sexuality as a human right in a digitalized world	Employee of the University of Hanover
8	Transformation of two clinics—moving from knowledge to action	Employee of the specialist clinic Gaißach, employee of the Charité Berlin
9	Duisburg-Essen University Hospital—Smart Hospital goes Green Hospital	Two employees of the Duisburg-Essen University Hospital
10	Digitization in agriculture and planetary health	JPE, employee from Health for Future
11	Ethical consideration of digital medicine and interoperability and equity in the context of digitization	Professor from Charité Berlin, professor from FOM University
12	Bar camp	Six sessions from various internal and external lecturers
13	Final quiz using Kahoot! [22]	JN, TSB, and JPE

a WT22/23: 2022-2023 winter term.

### Participants

#### Overview

Participation in the lecture series was open to everyone who was interested. Participants were made aware of the event through the curriculum, the social media channels of the university, the lecturers and experts, and various newsletters. A total of 697 participants were assessed for eligibility, since they had registered for the lecture series throughout the whole semester (including lecturers). An average of 257 (SD 33) participants attended each session and could also be invited to participate. About 91% (634/697) of all registered participants were students from 94 different universities and 98 different fields of study (eg, medicine, dentistry, psychology, and nursing, as well as theology, law, and business administration).

#### Sample Size

No formal a priori sample size calculation was conducted because the study was exploratory, and participation depended on voluntary attendance at the lecture series.

#### Recruitment

Recruitment materials were distributed through university mailing lists, social media channels, newsletters, and Zoom’s registration system. All individuals who registered for the lecture series via Zoom received email invitations to participate in the surveys, irrespective of university affiliation. Moodle announcements were also used for participants affiliated with the 2 participating universities. The procedure remained the same in both the T1 and T2 surveys.

Participants were recruited for the T1 survey from October 13, 2022, to October 28, 2022. The research project was introduced to all participants during the first session of DMgPH.

The link to the T1 survey was distributed twice via chat to all participants in the first session and, as described above, via email.

For the T2 survey, participants were recruited from January 19, 2023, to January 29, 2023. The T2-survey questionnaire was sent out on the penultimate and last days of the lecture series via the chat and, as described above, via email.

#### Eligibility Criteria

Participants were only able to take part in the survey if they answered that they were planning to attend the lecture series regularly (T1) and if they had attended the lecture series at least 11 of 13 times (T2). Additionally, they had to be of legal age (T1 and T2).

### Survey Instrument

#### Overview

An online questionnaire (Multimedia Appendix 1) was developed using the SoSciSurvey platform [23]. It was a nonexperimental, cross-sectional survey to explore group-level differences before and after the lecture series. All questions were to be answered voluntarily. During the T1 survey, participants were asked to generate a pseudonym, which they were asked to provide again during the T2 survey to merge T1 and T2 data. The pseudonym was derived by the participants from specific letters of personal information (first letter of the mother’s first name, last letter of the place of birth, birthday as a 2-digit number, first letter of the first name, and house number of the first address) to generate a test subject code (Multimedia Appendix 1).

The questionnaire itself consists of 4 sections. Section A assesses sociodemographic characteristics. Section B assesses ecological attitudes using the New Ecological Paradigm (NEP) scale, a validated instrument for measuring environmental worldviews and attitudes, which was used in its German translation. Sections C and D include items assessing both knowledge-related perceptions and attitudes toward climate-related health impacts, digitalization, and digital planetary health. These items were adapted from previous studies and translated using forward-backward translation where applicable. The feedback section includes a mix of knowledge- and attitude-related open-text responses regarding the lecture series and the integration of digital planetary health into university curricula.

The lecture series and questionnaire were informed by transformative learning theory, which provides a conceptual framework for understanding how learners engage with complex and value-laden topics such as digital planetary health. From this perspective, learning is not limited to the acquisition of factual knowledge but also involves critical reflection on existing assumptions and the integration of new perspectives into one’s understanding of the world [24,25]. However, whether such reflection or attitude-related differences translate into actual behavior change remains to be examined in future research.

The questionnaires at T1 and T2 were almost identical. Any discrepancies are explicitly noted below.

#### Sociodemographic Data

Sociodemographic information, including sex, age, size of household, school-leaving certificate, relationship to medical care, education by university, study program, and semester, was recorded as contextual data. In the T1 and T2 surveys, commitment to climate activist groups was assessed.

#### Relationship Between Humans and the Environment

This section examines the extent to which participants have an ecologically friendly and environmentally friendly worldview toward the health of the planet and how this is changing, on a scale ranging from 1 to 6 (1=“strongly disagree” to 6=“strongly agree”). The NEP scale, which is a validated instrument for assessing ecological worldviews [26], was used in its German translation [27].

#### Health Problem Assessment

The assessment of the participants toward digital planetary health is surveyed in this section. The survey is based on the list of various health problems used in other studies [28,29]. For the survey, the list was adapted and translated via forward-backward translation. The list was used to survey first whether participants are already experiencing effects from the climate catastrophe and second whether they suspect that this will be the case in 20 years (response options: “Yes”/“No”/“I don’t know”).

#### Attitude Toward Digitalization

In this section, the participants were asked for their opinions in free-text responses on how digitization can positively or negatively impact the climate crisis.

#### Attitude Toward Digital Planetary Health at the University

In T2, participants were asked to rate statements about the importance of addressing the climate catastrophe, its impact on health, and digital aspects in their own study program or other study programs, as well as their impressions of the scope of educational opportunities in the field of digital planetary health, which they rated on a scale from 1 to 6 (1=“do not agree at all” to 6=“completely agree”).

Participants were also asked to write down free-text responses on how topics related to digital planetary health could best be integrated into the curricula of study programs.

#### Feedback on the Lecture Series

Participants were also asked to give qualitative feedback on the lecture series at T2 only, using free-text responses (Textbox 1).

Textbox 1. **Content of the T1 and T2 surveys.**

- Sociodemographic data

-  Sex, age in years, size of household, school-leaving certificate, relationship to medical care (T1), education by university, study program, semester, commitment to climate activism (T1)

- Relationship between humans and the environment

-  Statements regarding the relationship between humans and the environment, based on the New Ecological Paradigm scale by Schleyer-Lindenmann et al [27] (15 statements ranging from 1 to 6)

- Health problem assessment

-  Rating of 12 health problems on whether they have increased at the current time, and rating of 12 health problems on whether they will increase in the next 20 years (response options: “Yes”/“No”/“I don’t know”)

- Attitude toward digitalization

-  Qualitative feedback on the influence of digitalization on digital planetary health

- Attitude toward digital planetary health at university

-  Importance of digital planetary health and perceptions of the sufficiency of existing educational opportunities of digital planetary health within participants’ own study programs or those of others (2 statements rated on a scale from 1 to 6), and qualitative feedback on opportunities to integrate digital planetary health into curricula

- Feedback on the lecture series

-  Qualitative feedback on the lecture series

### Data Analysis

#### Quantitative Analysis

The data analysis was primarily descriptive. First, datasets containing missing information were excluded from the analysis. A pseudonym-based comparison of responses between measurement points T1 and T2 was unsuccessful, meaning that no reliable longitudinal analyses could be carried out at the individual level. For this reason, the data from both measurement points were analyzed separately. The interpretation was based on the descriptive statistics obtained. Data analysis was performed using SPSS (version 29; IBM Corp).

#### Qualitative Analysis

The answers to the qualitative question in Section D were evaluated by content analysis [30] using MAXQDA Plus 2022 (VERBI Software GmbH) involving 2 researchers (TSB and IO). To begin with, the 2 researchers analyzed the entire dataset including answers to (1) attitude toward digitalization (T1 and T2), (2) attitude toward digital planetary health at universities (T2), and (3) feedback on the lecture series (T2).

Both researchers analyzed the data separately and inductively developed categories for sections A to C, resulting in 3 codebooks, one for each section. The 2 researchers met to discuss their findings and merged their coding systems. No conflicts arose that required the involvement of a third party. Intercoder agreement was not assessed. The quotes in the manuscript were selected to include particularly striking examples offering unique perspectives, as well as those reflecting a broad consensus and recurring frequently in a similar vein. Care was also taken to ensure a balanced representation of students and nonstudents, as well as across genders and degree programs to highlight the diversity of perspectives.

## Results

### Participant Profile

A total of 215 participants took part in the T1 survey, and 105 participants took part in the T2 survey. To ensure data quality, all incomplete data records were excluded during the data cleaning process. Only data records containing complete information on the relevant variables were included. A total of 180 participants from T1 and 79 participants from T2 were included in the final analysis (Table 2). The mean age in the T1 survey was 27.92 (SD 10.95) years, with a minimum age of 18 years and a maximum age of 77 years. The mean age in the T2 survey was 29.22 (SD 12.78) years, with a minimum age of 18 years and a maximum age of 78 years.

**Table 2.** Sociodemographic data of participants in the T1 and T2 surveys.

Characteristics	T1 (n=180)	T2 (n=79)
Age (y), mean (SD; range)	27.92 (10.95; 18-77)	29.22 (12.78; 18-78)
Gender, n (%)		
Women	137 (76.1)	52 (65.8)
Men	43 (23.9)	27 (34.2)
Student status, n (%)		
Student	155 (86.1)	68 (86.1)
Nonstudent	25 (13.9)	11 (13.9)
Household size, n (%)		
1	50 (27.8)	22 (27.8)
2	68 (37.8)	32 (40.5)
3	32 (17.8)	13 (16.5)
4	23 (12.8)	10 (12.7)
5	7 (3.9)	2 (2.5)
Currently working in the health care sector (y), n (%)		
Yes	83 (46.1)^a^	40 (50.6)^b^
No	97 (53.9)	39 (49.4)
Previously worked in the health care sector (y), n (%)		
Yes	35 (19.4)^c^	16 (20.3)^d^
No	145 (80.6)	63 (79.8)
Volunteering in climate protection, n (%)		
Little to no engagement	117 (65.0)	47 (59.5)
Moderate engagement	41 (22.8)	20 (25.3)
High engagement	22 (12.2)	12 (15.2)

a Mean 7.78 (SD 7.98; range 1-45).

b Mean 10.17 (SD 10.37; range 0.5-32).

c Mean 4.83 (SD 6.4; range 0.5-27).

d Mean 4.16 (SD 3.54; range 0.5-14).

The majority of participants were women. In the T1 survey (n=180), 137 (76.1%) participants were women and 43 (23.9%) participants were men. In the T2 survey (n=79), 52 (65.8%) participants were women and 27 (34.2%) participants were men. The “nonbinary” option was not selected.

In the T1 survey, 155 (86.1%) participants were students. Of those, 76.8% (119/155) were students at UW/H and 4% (6/155) were students at RUB. The remaining 19.4% (30/150) were from 13 other national and international universities. Twenty-five (13.9%) participants in the T1 survey were nonstudents.

In the T2 survey, 68 (86.1%) participants were students. About 86.8% (59/68) of them were at UW/H, 2.9% (2/68) at RUB, and 10.3% (7/68) at 3 other national universities (Table 2). In the T2 survey, 11 (13.9%) participants were nonstudents.

In terms of household size, most participants lived in 2-person households, with 68 (37.8%) participants in T1 and 32 (40.5%) participants in T2. Single-person households were the second most common category, comprising 50 (27.8%) participants in T1 and 22 (27.8%) participants in T2. Three-person households comprised 32 (17.8%) participants in T1 and 13 (16.5%) participants in T2. Four-person households were reported by 23 (12.8%) participants in T1 and 10 (12.7%) participants in T2. Households with 5 participants were only marginally represented, with 7 (3.9%) participants in T1 and 2 (2.5%) participants in T2.

At the time of the survey, 83 (46.1%) participants in T1 and 40 (50.6%) participants in T2 were working in the health care sector. In contrast, 97 (53.9%) participants in T1 and 39 (49.4%) participants in T2 were not working in the health care sector. The average length of professional experience in the health care sector among those currently employed was 7.78 (SD 7.98) years in T1 and 10.17 (SD 10.37) years in T2. In addition, 35 (19.4%) participants in T1 and 16 (20.3%) participants in T2 stated that they had previously worked in the health care sector.

As part of the T1 measurement, participants were asked whether they were already actively involved in climate protection (eg, in a Campus for Future activist group). This made it possible to estimate the participants’ handprints. One hundred seventeen (65%) participants in T1 and 47 (59.5%) participants in T2 reported low to no engagement. A moderate level of engagement was reported by 41 (22.8%) participants in T1 and 20 (25.3%) participants in T2. A high level of engagement was reported by 22 (12.2%) participants in T1 and 12 (15.2%) participants in T2.

### Relationship Between Humans and the Environment

The results of the T1 and T2 surveys using the NEP scale can be found in Table 3.

To examine differences between T1 and T2, descriptive statistics in the form of means and SDs were reported for individual items of the NEP scale (Table 3) and for the assessment of health problems (Table 4). Across most items, there were only minor differences between the 2 measurement points, with the means generally falling within comparable ranges and similar variation in responses.

**Table 3.** Content items regarding the New Ecological Paradigm scale in the T1 and T2 surveys^a^.

Item	T1 (n=180), mean (SD)	T2 (n=79), mean (SD)
We are approaching the limit of the number of people the Earth can support.	4.62 (1.32)	4.59 (1.45)
Humans have the right to modify the natural environment to suit their needs.	2.93 (1.14)	2.71 (1.14)
When humans interfere with nature it often produces disastrous consequences.	4.78 (1.10)	4.97 (1.05)
Human ingenuity will ensure that we do not make the Earth unlivable.	3.50 (1.18)	3.24 (1.22)
Humans are seriously abusing the environment.	5.22 (1.03)	5.34 (1.05)
The Earth has plenty of natural resources if we just learn how to develop them.	4.48 (1.29)	4.58 (1.31)
Plants and animals have as much right as humans to exist.	5.02 (1.20)	5.13 (1.23)
The balance of nature is strong enough to cope with the impacts of modern industrial nations.	2.15 (1.23)	2.03 (1.07)
Despite our special abilities, humans are still subject to the laws of nature.	5.04 (1.15)	5.03 (1.30)
The so-called “ecological crisis” facing humankind has been greatly exaggerated.	1.55 (0.97)	1.41 (0.91)
The Earth is like a spaceship with very limited room and resources.	3.58 (1.42)	3.59 (1.58)
Humans were meant to rule over the rest of nature.	1.72 (1.10)	1.53 (0.99)
The balance of nature is very delicate and easily upset.	4.57 (1.19)	4.53 (1.40)
Humans will eventually learn enough about how nature works to be able to control it.	2.90 (1.17)	2.76 (1.26)
If things continue on their present course, we will soon experience a major ecological catastrophe.	5.33 (1.03)	5.41 (1.01)

a Likert scale (1=“do not agree at all” and 6=“completely agree”).

**Table 4.** Content items regarding health problems at present and in 20 years in the T1 and T2 surveys^a^.

Item	T1 (n=180)		T2 (n=79)	
	Content items regarding health problems at present, mean (SD)	Content items regarding health problems in 20 years, mean (SD)	Content items regarding health problems at present, mean (SD)	Content items regarding health problems in 20 years, mean (SD)
Vector-borne infectious disease	0.99 (0.54)	0.91 (0.52)	1.03 (0.59)	0.86 (0.74)
Flooding-related displacement	0.94 (0.48)	0.98 (0.30)	0.91 (0.47)	0.99 (0.26)
Mental health conditions	1.00 (0.33)	0.88 (0.52)	0.99 (0.38)	1.00 (0.28)
Air quality–related illness	0.93 (0.67)	0.91 (0.55)	0.86 (0.63)	0.93 (0.50)
Food-borne disease	0.87 (0.92)	0.82 (0.78)	0.83 (0.92)	0.97 (0.67)
Disruption of health care services during extreme weather events	0.71 (0.96)	0.91 (0.58)	1.11 (0.70)	0.96 (0.60)
Water-borne infectious disease	0.66 (1.02)	0.89 (0.63)	0.84 (1.01)	0.91 (0.68)
Heat-related illness	0.88 (0.65)	0.91 (0.44)	0.93 (0.64)	0.96 (0.45)
Cold-related illness	0.88 (1.14)	0.69 (1.00)	0.86 (1.09)	0.92 (0.94)
Water-availability illness	0.83 (0.78)	0.95 (0.45)	0.91 (0.68)	0.93 (0.50)
Malnutrition	1.01 (0.67)	0.99 (0.50)	1.09 (0.57)	0.95 (0.57)
Other climate change health-related impacts	0.74 (0.75)	0.79 (0.63)	0.74 (0.74)	0.92 (0.48)

a Response options: “Yes (1)”/“No (0)”/“I don’t know” (2).

The descriptive statistics in Table 3 showed a broadly similar pattern of responses at both measurement points. Particularly high mean scores were found for the items “Humans are seriously abusing the environment” (T1: mean 5.22, SD 1.03; T2: mean 5.34, SD 1.05) and “If things continue on their present course, we will soon experience a major ecological catastrophe” (T1: mean 5.33, SD 1.03; T2: mean 5.41, SD 1.01). Comparatively high scores were also observed for “Plants and animals have as much right as humans to exist” (T1: mean 5.02, SD 1.20; T2: mean 5.13, SD 1.23) and for “Despite our special abilities, humans are still subject to the laws of nature” (T1: mean 5.04, SD 1.15; T2: mean 5.03, SD 1.33).

In contrast, the mean scores were lower for statements formulated in a more anthropocentric or techno-optimistic manner. This applied, for example, to “Human ingenuity will ensure that we do not make the Earth uninhabitable” (T1: mean 3.50, SD 1.19; T2: mean 3.24, SD 1.22) and “Humans were meant to rule over the rest of nature” (T1: mean 1.72, SD 1.10; T2: mean 1.54, SD 1.00). The item “The so-called ecological crisis facing humankind has been greatly exaggerated” also showed comparatively low levels of agreement (T1: mean 1.55, SD 0.97; T2: mean 1.41, SD 0.91).

The descriptive statistics in Table 4 showed a broadly similar pattern of responses for both T1 and T2. For the assessment of current health problems, the mean scores at both measurement points were predominantly in the range of approximately 0.66 to 1.01. Particularly high values were observed, for example, for mental health conditions, with mean 1.00 (SD 0.33) in T1 and mean 0.99 (SD 0.38) in T2, as well as for malnutrition, with mean 1.01 (SD 0.67) in T1 and mean 1.09 (SD 0.57) in T2. Comparatively lower mean values were reported, among other things, for water-borne infectious diseases, which stood at mean 0.66 (SD 1.02) in T1 and mean 0.84, SD (1.01) in T2.

Similar values were also observed across both measurement points for the assessment of health problems in 20 years. Here, the mean values generally ranged between 0.79 and 0.99. For example, the mean for mental health conditions was 0.88 (SD 0.52) in T1 and mean 1.00 (SD 0.28) in T2. Malnutrition also showed relatively high values, with mean 0.99 (SD 0.50) in T1 and mean 0.95 (SD 0.57) in T2. By contrast, slightly lower mean values were observed for food-borne disease, with mean 0.82 (SD 0.78) in T1 and mean 0.97 (SD 0.67) in T2, as well as for other climate change–related health impacts, with mean 0.79 (SD 0.63) in T1 and mean 0.92 (SD 0.48) in T2.

Overall, the descriptive values suggested that respondents assessed the various health impacts of climate change to a similar extent, both currently and in the future. The SDs varied across the items but were moderate overall, indicating a certain degree of variation within the sample. Descriptively, there were only minor differences between T1 and T2.

### Health Problems

The results from T1 and T2 regarding the assessment of health problems at the time of the survey and in 20 years’ time are shown in Table 4.

#### Attitude Toward Digitalization

During the qualitative evaluation of the answers to this section, 290 codes were identified, with positive evaluations spanning 197 codes and negative evaluations of digitalization spanning 53 codes. In addition, ambiguous evaluations (21 codes) and demands (19 codes) were identified.

Participants described digitalization predominantly as a tool with transformative potential for addressing the climate crisis and strengthening planetary health. Responses frequently reflected a perceived tension between the transformative potential of digital technologies and concerns regarding their environmental and societal consequences.

A central theme concerned the ability of digital technologies to facilitate communication, collaboration, and access to information. Participants associated digitalization with faster dissemination of knowledge, improved networking across disciplines and regions, and lower barriers to participation, particularly for people in rural areas or with limited access to educational resources. Participants further described digital technologies as supporting organizational efficiency, crisis management, and broader transformation processes within society and health care.

Within the health care context, participants particularly emphasized the potential of digital technologies to improve access to care independent of location. In addition, they referred to opportunities for more efficient collaboration, prevention, diagnostics, and therapy, as well as the reduction of duplicated services and unnecessary resource use. Several participants further emphasized the potential of digital technologies to support public education and global engagement in planetary health.

At the same time, participants stressed that technological innovation alone would not be sufficient and repeatedly linked digitalization to broader societal transformation processes and normative questions of responsibility. One participant stated, “We need to transform our society(s) and change our own attitudes in the process” (T1: student of sustainability management).

Participants also emphasized that the societal benefits of digitalization depend on ethical and sustainable implementation. One participant stated, “However, this requires prudent planning, honesty and innovation for the benefit of the people and not greed for profit” (T2: psychology student).

Alongside the opportunities, participants critically reflected on the ecological and social risks associated with digitalization. Most prominently, they referred to increasing resource consumption, including electricity, rare-earth extraction, and electronic waste. In addition, participants raised serious concerns regarding negative impacts on mental health, dependency on technology, social isolation, and ethical risks such as discrimination by AI.

#### Digital Planetary Health in Study Programs

Participants largely regarded digital planetary health as an important topic for higher education and emphasized the need for stronger curricular integration across study programs. While respondents perceived the topic as highly relevant, they simultaneously described existing educational opportunities in this field as insufficient.

The results of the survey among T2 participants on the relevance of teaching digital planetary health are presented in Table 5.

**Table 5.** Attitudes toward digital planetary health at university in the T2 survey (n=79)^a^.

Item	Mean (SD)	Participants with partial to full agreement, n (%)
It is important to address climate catastrophe, health impacts, and digital issues in the curriculum.	5.38 (1.16)	56 (70.9)
I have the impression that students (at my university) receive sufficient educational offerings in the area of digital planetary health.	3.49 (1.47)	38 (48.1)

a Likert scale (1=“do not agree at all” to 6=“completely agree”).

In addition to the quantitative query, survey participants were asked at T2 to describe how topics related to digital planetary health could best be integrated into their own studies and/or courses with which they were familiar. A total of 60 codes were identified in the qualitative analysis.

In the qualitative responses, participants repeatedly expressed the wish that educational offerings on digital planetary health should become more broadly accessible across universities and disciplines. Many participants advocated permanent integration into curricula, including both mandatory and elective formats. Mandatory teaching was perceived as particularly necessary to ensure that the topic reaches students beyond those already interested in sustainability-related issues. One participant stated, “[...] otherwise only the engaged ones learn it, which are only a fraction of all students” (T2: medical student).

Participants further described digital planetary health education as an opportunity to develop new perspectives on familiar topics and to strengthen their perceived ability to contribute to societal change (“handprint”). Practical relevance and applicability to future professional contexts were repeatedly emphasized.

Moreover, respondents highlighted the value of multiprofessional collaboration and interactive teaching formats. They emphasized that the lecturers should possess strong expertise in the field and communicate content in an evidence-based yet emotionally engaging manner. Participants also expressed interest in more specialized content, including preventive medicine and health psychology.

### Feedback

A total of 78 codes were identified in the qualitative analysis of this section.

Overall, participants described the lecture series positively and highlighted both the interdisciplinary perspective and the digital format as particular strengths.

Participants frequently characterized the sessions as interactive, engaging, and informative. The atmosphere and moderation were described as pleasant, while the invited speakers were perceived as competent and knowledgeable. Participants particularly appreciated the combination of digital health and planetary health perspectives, which many had not previously encountered in an integrated educational format:

*It was super interesting, the linkages between Planetary Health and Digital Medicine have never really been presented in such a combined way. Thank you for offering this lecture series.*

[T2: medical student]

The digital format was also perceived as increasing flexibility and enabling exchange among participants and experts from diverse backgrounds. Participants valued the broad overview of the field and described the lecture series as successfully connecting prior knowledge with new perspectives.

Beyond knowledge acquisition, several participants reported that the lecture series stimulated personal reflection and discussions within their social environments regarding the climate crisis and environmental responsibility:

*[…] the event made me reflect on my own environmental behavior and engage in discourse and exchange with friends/family on such topics. […]*

[T2: health science student]

Critical feedback mainly focused on the desire for greater inclusion of topics related to animals and plants, more opportunities for participants’ own research activities, and, in some cases, a more neutral stance from lecturers.

Participants also suggested expanding interactive and research-oriented elements in future iterations of the lecture series.

## Discussion

### Principal Findings

This study explored participants’ perceptions, attitudes, and knowledge related to digital planetary health before and after participation in a fully digital lecture series. Overall, the findings suggest a high perceived relevance of integrating digital planetary health into higher education curricula. Participants particularly emphasized the importance of addressing climate-related health impacts within university education and highlighted the potential of digital technologies. The survey responses from T1 and T2 further indicate that participation in the lecture series may have contributed to increased awareness of specific climate-related health risks, particularly regarding water- and food-borne diseases. At the same time, qualitative findings revealed a differentiated understanding of digitalization, as participants described both opportunities and challenges associated with digital transformation in relation to sustainability.

In their feedback on the lecture series, the participants stated that the lecture series was positive and encouraged them to reflect on their own behavior and talk about it with friends and family. This finding is particularly important in the training of researchers, as the body of research on the twin transformation, sustainability, and digitalization in the health care sector is rather limited [31]. The survey results underline the relevance of interdisciplinary educational approaches that connect planetary health, digital health, and societal transformation. Universities do not merely have a duty to highlight problems; they must also make their own efforts, for example, by operating within planetary boundaries [32]. McGeown and Barry [33] put it in strong terms: “As producers and gatekeepers of knowledge, and as providers of education and training, our universities play a key role in the reproduction of unsustainability.” To overcome this, it seems sensible to develop new and open formats that bring students and society together to discuss problems and solutions.

The results of the T1 survey show that the participants started the lecture series with a high level of knowledge and awareness of the climate crisis. This self-selection bias has been identified in various studies and must be explicitly addressed and overcome through curriculum planning [34]. For future similar formats, it is of great relevance to reach people who are also active outside this focus and who have been less involved in the field of planetary health to date, for example, by incorporating it directly into the compulsory curriculum of the degree programs. This is consistent with the data collected on engagement in climate protection among the participants in the lecture series: an extrapolation to the whole population in Germany aged 14 years and older, based on a survey of 23,524 participants, reveals that 23% of the population is engaged in climate protection [35]. Of the 180 participants, 63 (35%) in the T1 survey stated that they are engaged in climate protection groups at a moderate or high level.

Another challenge here is that some prior knowledge was necessary to be able to follow the event fully. Both nationally in Germany [36] and internationally [37,38], there are already various educational offerings in the field of planetary health for HCPs. It is now crucial to network these programs, create synergies, and channel existing activism. This requires strategically oriented education, supported by integration into curricula, to increase awareness and reach people who are not yet aware of the issue. It could also be worth considering advertising the event on social media, in print media (daily newspapers), and on the radio. Particularly at a time when AI is being used more and more, the call for a digital planetary health perspective is becoming increasingly urgent [39]. It is desirable that this be implemented jointly by academia and society in the form of transformative science [40].

With regard to the selection of guest lecturers, it should be noted that they were recruited from the lecturers’ network. The involvement of guest experts in lectures ensures a good balance between theory and practice, thereby fostering both a love of learning and the application of knowledge [41]. While there was a lack of remuneration, lecturers gained value through interaction with the participants. To prevent excessive subjectification, a meeting with the lecturers and guest lecturers took place beforehand, and a guideline on scientific working methods and gender-sensitive formulations was provided. The possibility of offering a wide variety by inviting experts in combination with its own sessions was judged to outweigh the risk of subjectification in the previous consideration. In addition, the discussion offered the opportunity to organize the content at any time. It has also become apparent in other fields that the multiperspective approach offered by various guest lecturers leads to holistic learning success among lecture attendees [42].

From a behavioral psychology perspective, the changes observed in participants following a lecture series such as this can be explained by several mechanisms. For instance, a review by Dolcos et al [43] shows that emotions, or emotionally charged content, can significantly enhance both attention and episodic long-term memory. Building on this, Immordino-Young and Damasio [44] emphasize that the close link between emotion and cognition, which is continuously at work in the processing of external stimuli, forms a central basis for moral and ethical judgment.

Against this backdrop, the lecture series focused thematically on future prospects and the risks of the climate crisis. At the same time, the program was deliberately designed to highlight not only problem areas but also concrete solutions and individual opportunities for action to strengthen a sense of collective efficacy. This approach aligns with the guiding principles of Education for Sustainable Development, which aim to empower people to actively shape sustainable transformation processes. The results indicate possible starting points for these changes, but they should be viewed as trends.

A clear example of the link described above is provided by the third session, in which the German Sustainability Code and its practical implementation were presented. Overall, the lecture series thus aims to foster students’ moral judgment and values through engagement with ethical issues. At the same time, they are encouraged to develop their own positions and to critically engage with different perspectives [45].

In addition to the individual options for action identified, a dynamic also developed during the lecture series that led to further synergies. Several ideas for qualification projects have emerged and are being actively worked on today [46]. There have been various invitations and opportunities at conferences to present the lecture series and engage in discussions about it [47]. In December 2023, the team received the 1st Humboldtⁿ Prize for Sustainability ( 1. Humboldtⁿ-Preis für Nachhaltigkeit) for the lecture series itself. A concept for a lecture series on human-animal relationships was developed based on course discussions and implemented in subsequent semesters to revisit the many facets of this topic. Accompanying research was also carried out, focusing particularly on changes in participants’ attitudes and knowledge [48,49].

### Limitations

When looking at the results, several limitations should be considered. First, it is important to note that 86.1% (155/180) of the participants in the T1 survey were students. Opening up the event and promoting it to the wider community proved challenging during implementation. While students from various universities were attracted, the proportion of citizens as participants was very low. In addition, more people from the field of health care were reached. The target group of the lecture series—(future) HCPs—consists of trusted contact persons for their patients and communities. They are, therefore, an important and critical target group to inform and educate about the complex dynamics of climate change [50]. Second, participation in the lecture series was voluntary, so self-selection bias is likely, as individuals already interested in planetary health, sustainability, or digital medicine may have been more likely to participate. Third, reliable longitudinal matching between T1 and T2 was not possible, thereby limiting the assessment of a causal effect of the lecture series. Moreover, the study lacked a control group, which limits causal interpretation. Possible ceiling effects due to high baseline awareness, as well as social desirability in self-reported responses, may also have influenced the findings.

Future surveys should track whether students also take advantage of other learning opportunities on the topics of planetary health and digital medicine outside of the lecture series during the survey period.

### Conclusions

This study shows that attendees of the digital lecture series perceived digital planetary health as highly relevant to university education. The lecture series was positively received, particularly regarding its interdisciplinary perspective, digital accessibility, and the integration of planetary health and digital medicine. The comparison of T1 and T2 responses showed exploratory group-level differences in selected perceptions of climate-related health impacts. These findings support the importance of further integrating digital planetary health into university teaching.

## Supplementary material

10.2196/89259 Multimedia Appendix 1 Online questionnaire (English translation): T1 and T2 surveys.

## Abbreviations

DMgPH: Digital Medicine goes Planetary Health
HCP: health care professional
NEP: New Ecological Paradigm
RUB: Ruhr University Bochum
UW/H: Witten/Herdecke University
