# Correction: Attitudes Toward Digital Planetary Health Before and After a Lecture Series on Environmental Awareness and Future Visions: Repeated Cross-Sectional Survey Study Regarding Twin Transformation

**DOI:** 10.2196/111087

**Published:** 2026-09-25

**Authors:** Julia Nitsche, Theresa Sophie Busse, Sonja Schmalen, Ina Otte, Jan Peter Ehlers

**Affiliations:** 1 Department of Didactics and Educational Research in Health Science, Faculty of Health, Witten/Herdecke University, Alfred-Herrhausen-Straße 50, Witten, North Rhine-Westphalia, 58455, Germany, 49 02302 926 ext 7; 2 Junior Professorship for Digital Health, Faculty of Health, Witten/Herdecke University, Witten, North Rhine-Westphalia, Germany; 3 Health for Future, Deutsche Allianz für Klimawandel und Gesundheit, Berlin, Germany; 4 Department for Health Care Research, Institute for Diversity Medicine, Ruhr University Bochum, Bochum, North Rhine-Westphalia, Germany

In “Attitudes Toward Digital Planetary Health Before and After a Lecture Series on Environmental Awareness and Future Visions: Repeated Cross-Sectional Survey Study Regarding Twin Transformation” [1], the authors noted three errors.

The degree for author TSB was changed from the following:

*Dr rer medic*

The degree now reads:

*Prof Dr rer medic*

In Table 1, the following text in the first row has been revised:

*JN, TSB, SSchS*

The text now reads:

*JN, TSB, SSch*

Finally, in the Background section, the following sentence was revised:

*Lecturers from Witten/Herdecke University (UW/H; JN, TSB, and JPE), German Climate Change and Health Alliance ( SSch), and Ruhr University Bochum (RUB; IO)*

The sentence now reads:

*Lecturers from Witten/Herdecke University (UW/H; JN and JPE), German Climate Change and Health Alliance (SSch), and Ruhr University Bochum (RUB; TSB)*

The corrections will appear in the online version of the paper on the JMIR Publications website together with the publication of this correction notice. Because this was made after submission to PubMed, PubMed Central, and other full-text repositories, the corrected article has also been resubmitted to those repositories.
