# Supplementary material for: Attitudes Toward Digital Planetary Health Before and After a Lecture Series on Environmental Awareness and Future Visions: Repeated Cross-Sectional Survey Study Regarding Twin Transformation
Source: JMIR Med Educ. 2026 Aug 4;12:e89259. doi: 10.2196/89259 (PMC13436795; doi:10.2196/89259)
Supplement: Supplementary file 1 [file mededu-v12-e89259-s001.docx]

Appendix A - Attitudes Toward Digital Planetary Health Before and After a Lecture Series on Environmental Awareness and Visions of the Future - A repeated Cross-Sectional Survey Study regarding Twin-Transformation

**T1 Survey**

**Conditions for participation**

You can only participate in this study if you are of legal age. Are you already of legal age?

□ I am of legal age	□ I am not of legal age

**Participation**

Do you plan to take part in the "Digital Medicine goes Planetary Health" course regularly? □ Yes	□ No

## Sex

□ Female	□ Male	□ Diverse/Non-binary	□ Other: ______________

**Age in years**

_______

## Size of household

With how many people do you live in a household (1 = I live alone)? __________

## School-leaving certificate

## Highest general School-leaving qualification

□ I left school without completing secondary school

□ I left school with a general certificate of secondary education (5 years secondary school)

□ I left school with a general certificate of secondary education (6 years secondary school)

□ I have completed the 10th grade of polytechnic secondary school (before 1965: 8th grade)

□ I have the college entrance qualification

□ I have the general or subject-specific college and university entrance qualification (grammar school or EOS)

□ I have another school-leaving certificate: __________

**Relation to medical care:**

□ I am working in the healthcare sector

If yes: since how many years? __________

□ I have worked in the healthcare sector, but no longer.

If yes: for how many years __________

## Education by university

I am currently studying at the following university:

□ Witten/Herdecke University	□ Ruhr University Bochum	□ Other: __________

**Study program**

I am currently studying in the following study program:

□ Human medicine	□ Dentistry	□ Philosophy, politics and economics (B.A.)

□ Management (B.Sc.)	□ General Management (M.A.)

□ Psychology / psychotherapy (B.Sc.)	□ Clinical psychology / psychotherapy (M.Sc.)

□ Community Health Nursing (M.Sc.)	□ Ethics and organisation (M.A.)

□ Nursing Science (M.Sc.)	□ Other____________________

**Semester**

I am currently in the following semester:

______________	□ not applicable

**Commitment**

I am actively committed to climate protection, for example in a campus for future activists group. (1=do not agree at all; 6=completely agree)

□ 1	□ 2	□ 3	□ 4	□ 5	□ 6

**The relationship between humans and the environment**

## Below are a number of statements regarding the relationship between humans and the environment (Schleyer, Lindenmann et al., 2018). For each statement, please indicate the extent to which you agree with it. (1=strongly disagree, 6=strongly agree)

|  | 1 | 2 | 3 | 4 | 5 | 6 |
| --- | --- | --- | --- | --- | --- | --- |
| We are approaching the limit of the population that the earth can cope with. |  |  |  |  |  |  |
| People have the rights to change their natural environment in order to adapt it to their needs |  |  |  |  |  |  |
| When people interfere into nature, the consequences are often catastrophic. |  |  |  |  |  |  |
| Human ingenuity will ensure that we do NOT make the earth uninhabitable. |  |  |  |  |  |  |
| People abuse the environment badly. |  |  |  |  |  |  |
| The earth has many natural resources, if we learn to promote them. |  |  |  |  |  |  |
| Plants and animals have the same rights to live as humans. |  |  |  |  |  |  |
| The equilibrium of the nature is strong enough, to cope with the influence of modern industrialized nations. |  |  |  |  |  |  |
| Apart from our special abilities, we humans are still subject to the laws of nature. |  |  |  |  |  |  |
| The so-called "environmental crisis" that humanity is facing is completely exaggerated. |  |  |  |  |  |  |
| Earth is like a spaceship with little space and just a few resources. |  |  |  |  |  |  |
| Humans were born to rule over the rest of nature. |  |  |  |  |  |  |
| The equilibrium of nature is very delicate and easily disturbed. |  |  |  |  |  |  |
| Humans may learn enough about how nature works to be able to control it. |  |  |  |  |  |  |
| If things continue as they are, we will soon experience a major environmental catastrophe. |  |  |  |  |  |  |

**Health problems**

## The following list includes 12 health problems. Please estimate whether the problem has increased at the current time.

|  | Yes | No | I don’t know |
| --- | --- | --- | --- |
| Infectious diseases |  |  |  |
| Flood-related displacement |  |  |  |
| Mental diseases |  |  |  |
| Diseases related to air quality |  |  |  |
| Foodborne diseases |  |  |  |
| Interruptions to healthcare during extreme weather events |  |  |  |
| Water-borne infectious diseases |  |  |  |
| Heat-related diseases |  |  |  |
| Cold-related diseases |  |  |  |
| Water-availability related diseases |  |  |  |
| Malnutrition |  |  |  |
| Other health-related effects of climate change |  |  |  |

## Health problems II

## Now please estimate whether the problem will increase in the next twenty years.

|  | Yes | No | I don’t know |
| --- | --- | --- | --- |
| Infectious diseases |  |  |  |
| Flood-related displacement |  |  |  |
| Mental diseases |  |  |  |
| Diseases related to air quality |  |  |  |
| Foodborne diseases |  |  |  |
| Interruptions to healthcare during extreme weather events |  |  |  |
| Water-borne infectious diseases |  |  |  |
| Heat-related diseases |  |  |  |
| Cold-related diseases |  |  |  |
| Water-availability related diseases |  |  |  |
| Malnutrition |  |  |  |
| Other health-related effects of climate change |  |  |  |

**Digitalization**

How do you think digitalization can influence this positively or negatively?

Write as much or as little as you like.

**T2 Survey**

**Conditions for participation**

You can only participate in this study if you are of legal age. Are you already of legal age?

□ I am of legal age	□ I am not of legal age

**Participation**

Did you take part regularly in the "Digital Medicine goes Planetary Health" course (at least 11 of the 13 sessions)?

□ Yes	□ No

## Sex

□ Female	□ Male	□ Diverse/Non-binary	□ Other: ______________

**Age in years**

_______

## Size of household

With how many people do you live in a household (1 = I live alone)? __________

## School-leaving certificate

## Highest general School-leaving qualification

□ I left school without completing secondary school

□ I left school with a general certificate of secondary education (5 years secondary school)

□ I left school with a general certificate of secondary education (6 years secondary school)

□ I have completed the 10th grade of polytechnic secondary school (before 1965: 8th grade)

□ I have the college entrance qualification

□ I have the general or subject-specific college and university entrance qualification (grammar school or EOS)

□ I have another school-leaving certificate: __________

**Relation to medical care:**

□ I am working in the healthcare sector

If yes: since how many years? __________

□ I have worked in the healthcare sector, but no longer.

If yes: for how many years __________

## Education by university

I am currently studying at the following university:

□ Witten/Herdecke University	□ Ruhr University Bochum	□ Other: __________

**Study program**

I am currently studying in the following study program:

□ Human medicine	□ Dentistry	□ Philosophy, politics and economics (B.A.)

□ Management (B.Sc.)	□ General Management (M.A.)

□ Psychology / psychotherapy (B.Sc.)	□ Clinical psychology / psychotherapy (M.Sc.)

□ Community Health Nursing (M.Sc.)	□ Ethics and organisation (M.A.)

□ Nursing Science (M.Sc.)	□ Other____________________

**Semester**

I am currently in the following semester:

______________	□ not applicable

**Commitment**

I am actively committed to climate protection, for example in a campus for future activists group. (1=do not agree at all; 6=completely agree)

□ 1	□ 2	□ 3	□ 4	□ 5	□ 6

**The relationship between humans and the environment**

## Below are a number of statements regarding the relationship between humans and the environment (Schleyer, Lindenmann et al., 2018). For each statement, please indicate the extent to which you agree with it. (1=strongly disagree, 6=strongly agree)

|  | 1 | 2 | 3 | 4 | 5 | 6 |
| --- | --- | --- | --- | --- | --- | --- |
| We are approaching the limit of the population that the earth can cope with. |  |  |  |  |  |  |
| People have the rights to change their natural environment in order to adapt it to their needs |  |  |  |  |  |  |
| When people interfere into nature, the consequences are often catastrophic. |  |  |  |  |  |  |
| Human ingenuity will ensure that we do NOT make the earth uninhabitable. |  |  |  |  |  |  |
| People abuse the environment badly. |  |  |  |  |  |  |
| The earth has many natural resources, if we learn to promote them. |  |  |  |  |  |  |
| Plants and animals have the same rights to live as humans. |  |  |  |  |  |  |
| The equilibrium of the nature is strong enough, to cope with the influence of modern industrialized nations. |  |  |  |  |  |  |
| Apart from our special abilities, we humans are still subject to the laws of nature. |  |  |  |  |  |  |
| The so-called "environmental crisis" that humanity is facing is completely exaggerated. |  |  |  |  |  |  |
| Earth is like a spaceship with little space and just a few resources. |  |  |  |  |  |  |
| Humans were born to rule over the rest of nature. |  |  |  |  |  |  |
| The equilibrium of nature is very delicate and easily disturbed. |  |  |  |  |  |  |
| Humans may learn enough about how nature works to be able to control it. |  |  |  |  |  |  |
| If things continue as they are, we will soon experience a major environmental catastrophe. |  |  |  |  |  |  |

**Health problems**

## The following list includes 12 health problems. Please estimate whether the problem has increased at the current time.

|  | Yes | No | I don’t know |
| --- | --- | --- | --- |
| Infectious diseases |  |  |  |
| Flood-related displacement |  |  |  |
| Mental diseases |  |  |  |
| Diseases related to air quality |  |  |  |
| Foodborne diseases |  |  |  |
| Interruptions to healthcare during extreme weather events |  |  |  |
| Water-borne infectious diseases |  |  |  |
| Heat-related diseases |  |  |  |
| Cold-related diseases |  |  |  |
| Water-availability related diseases |  |  |  |
| Malnutrition |  |  |  |
| Other health-related effects of climate change |  |  |  |

## Health problems II

## Now please estimate whether the problem will increase in the next twenty years.

|  | Yes | No | I don’t know |
| --- | --- | --- | --- |
| Infectious diseases |  |  |  |
| Flood-related displacement |  |  |  |
| Mental diseases |  |  |  |
| Diseases related to air quality |  |  |  |
| Foodborne diseases |  |  |  |
| Interruptions to healthcare during extreme weather events |  |  |  |
| Water-borne infectious diseases |  |  |  |
| Heat-related diseases |  |  |  |
| Cold-related diseases |  |  |  |
| Water-availability related diseases |  |  |  |
| Malnutrition |  |  |  |
| Other health-related effects of climate change |  |  |  |

**Digitalization**

How do you think digitalization can influence this positively or negatively?

Write as much or as little as you like.

**Digital Planetary Health in study programs**

Below you will find some statements on the relationship to the relevance of Digital Planetary Health to your study program or the study programs of others in terms of the general orientation of current study programs. For each statement, please indicate the extent to which you agree (1= do not agree at all, 6= completely agree).

|  | 1 | 2 | 3 | 4 | 5 | 6 |
| --- | --- | --- | --- | --- | --- | --- |
| It is important to address the climate catastrophe, the impact on health and digital aspects during the study program. |  |  |  |  |  |  |
| I have the impression that students (at my university) receive sufficient educational opportunities in the field of Digital Planetary Health. |  |  |  |  |  |  |

Please write down how the topics related to Digital Planetary Health could best be integrated into the curriculum of your study program(s).

Write as much or as little as you like.

**Feedback**

We welcome feedback (both positive and negative) on the course ‘Digital Medicine goes Planetary Health’. Please feel free to share your thoughts in the comments section.
